# The effect of vinegar and drying (Solar and Open Sun) on the microbiological quality of ginger (*ZINGIBER OFFICINALE ROSCOE*) rhizomes

**DOI:** 10.1002/fsn3.1902

**Published:** 2020-09-19

**Authors:** Roseline Esi Amoah, Sureshkumar Kalakandan, Faustina D. Wireko‐Manu, Ibok Oduro, Firibu Kwesi Saalia, Ebenezer Owusu

**Affiliations:** ^1^ Ghana Standards Authority Accra Ghana; ^2^ Department of Food Biotechnology Indian Institute of Food processing Technology (IIFPT) Thanjavur Tamil Nadu India; ^3^ Department of Food science and Technology Kwame Nkrumah University of Science and Technology Kumasi Ghana; ^4^ ^4^Department of Food Process Engineering University of Ghana Legon Ghana; ^5^ Department of Plant and Environmental Biology University of Ghana Legon Ghana

**Keywords:** Ginger, Microbial load, Rhizomes, Solar drying, Vinegar

## Abstract

This study evaluated the influence of 10% vinegar and solar drying using two solar dryers and open‐sun drying on the microbiological quality of ginger (*Zingiber officinale* Roscoe) rhizome. The rhizomes were analyzed for bacterial, mold, and *Salmonella* populations in the raw state, which were water‐washed and soaked in 10% vinegar, and in dried form. The fungal population was isolated and identified. Fresh and dried ginger rhizome contained both bacterial and fungal population in the range of 3.0 x 10^2^ ± 1.14 x 10^2^ to 2,180 x 10^9^ ± 70.7 x 10^9^ CFU/g. The stainless steel solar dryer had fewer fungal loads among the drying methods. *Aspergillus* and *Penicillium* species of mycotoxin‐producing potential were identified. The 10% vinegar as pretreatment showed no significant difference (*p* ≤ .05) in the bacterial population reduction but in the fungal population reduction. Growth of fungi in fresh and dried ginger extracts was lower compared with growth in Potato Dextrose Broth.

## Graphical abstract

Vinegar‐pretreated fresh ginger was associated with fungi from the genus *Aspergillus, Penicillium, Mucor, and Rhizopus*. Drying using the solar dryers reduced microbial load of the ginger.

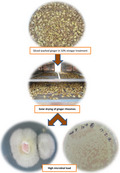

## 1 INTRODUCTION

Ginger (*Zingiber officinale* Roscoe) is the rhizome of the Zingiberaceae family used as a spice and herbal medicine (FAO, 2002; Gupta & Tennyson, 2020; Mohammed et. al., 2019). It can be obtained worldwide in its fresh or semiprocessed form. Spices and culinary herbs have always been part of the diet since ancient times, and indeed have been a major part of merchandize during early global trade and voyages. With the increasing awareness of the association between dietary phytochemicals and human health and well‐being, spices and culinary herbs have gained the attention of the scientific community because they contain chemical compounds that possess antioxidants and medicinal properties (Jeswal & Kumar, 2015; Madsen et al., 1995; Mohammed et. al., 2019; Sharma et. al., 2017). These findings, together with the functionality of culinary herbs and spices in improving the flavor and consumer appeal of diets, have led to increased application in a wider variety of savory foods. The rising popularity of culinary herbs and spices, however, may come with safety concerns depending on the mode of usage and the severity of heat treatment before consumption. Culinary herbs and spices can harbor pathogenic microorganisms if not properly processed or adequately pretreated to eliminate them.

The microflora of black pepper, red pepper, white pepper, cumin, coriander, allspice, and ginger in Turkey revealed the prevalence of *S. aureus, E. coli, B.cereus, and Salmonella sp*. (Hamparsun et. al, 2009). Studies on the mycoflora of ginger in Egypt and several spices and medicinal herbs in Iraq and India revealed the dominant isolated fungi as belonging to the genera *Aspergillus, Penicillium, Alternaria, Absidia, Cladosporium and Rhizopus* (Abdulkadir et. al., 2002; Aziz et. al., 1998; Chourasia, 1995; Farid et. al., 2013; Geeta & Reddy, 1990; Jeswal & Kumar, 2015; Rani et.al., 1995; Tainter & Grenis, 1993, 1994). Ramesh and Santoshkumar (2013) also worked on several samples of pepper, coriander, cumin, cardamom, and India cassia from the open market in India isolated fungi belonging to 44 genera of which the most dominant were *Absidia, Mucor*, *Rhizopus, Aspergillus, Cladosporium, curvularia, Penicillium*, *Alternaria,* and *Fusarium* species. Yet, other studies by Jeswal and Kumar (2015) and Punam and Dhiraj (2015) from the same country to assess the natural occurrence of toxigenic mycoflora and ochratoxin A in a wide range of spices showed *A. flavus, A. niger, A. parasiticus* and *Penicillium citrinum* as the dominant species. Sumanth and Waghmare (2010) working on the seed mycoflora of spices also isolated *Alternaria, Aspergillus, Cladosprium, Curvularia, Fusarium, Helminthosporium, and Trichoderma* as the dominant genera. In Ghana, studies by Addo (2005) and Ahene et al. (2011) have shown that some spices and dehydrated products are contaminated with pathogenic bacteria such as *Enterobacter cloacae*, *Enterobacter sakazaki, Aeromonas salmonicida,* and *Chromobacterium violaceum*. Addo (2005) also showed that ginger was heavily contaminated with fungi. Thus, while there may be increased consumption of ginger because of the growing awareness of its health benefits, this might paradoxically expose consumers to microbiologically contaminated product if not adequately sanitized and processed.

To minimize microbial load, and extend the shelf life of many agricultural products, pretreatments (Arserim‐Uçar & Çabuk, 2020) together with drying technology are used for food preservation. In most developing countries with abundant sun energy, solar drying could be an advantage over the open‐sun drying to reduce microbial contamination, which is associated with the open‐sun drying (Deshmukh et. al., 2014). There are several chemicals to pretreat the ginger to reduce the microbial load yet consumers would prefer chemical‐free dried ginger. Vinegar is a friendly household chemical that is mostly used to sanitize fruits and vegetables.

Ginger may be cooked in some food applications as an ingredient and will therefore be microbiologically safe. On the other hand, it is also used without heat treatment in certain applications, and that might be hazardous to consumers. This study investigated the effects of pretreatment with vinegar, before solar drying or open‐sun drying, on the microbiological load of ginger rhizomes.

## 2 MATERIALS AND METHODS

### 2.1 Source of raw materials

The yellow variety of fresh ginger rhizome (*Zingiber officinale* Roscoe) was purchased from the open market in Agbogbloshie, Accra Ghana. The fresh ginger rhizome was transported to the laboratory of Ghana Standards Authority in jute sack and hand was cleaned of excess debris. It was divided into two parts; one part remained in Ghana, and the other one part was couriered to India Institute of Food Processing Technology (IIFPT) of Ministry of Food and Processing, India (MOFPI), Thanjavur, Tamil Nadu.

### 2.2 Study location

**Figure 1:**
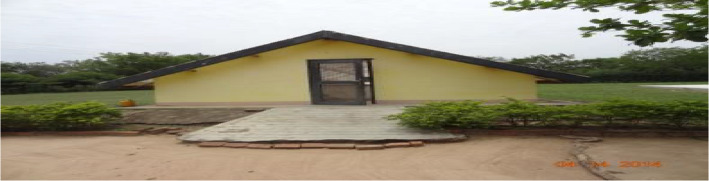
Pictorial representation of the tent‐like concrete solar dryer (CSD)

**Figure 2:**
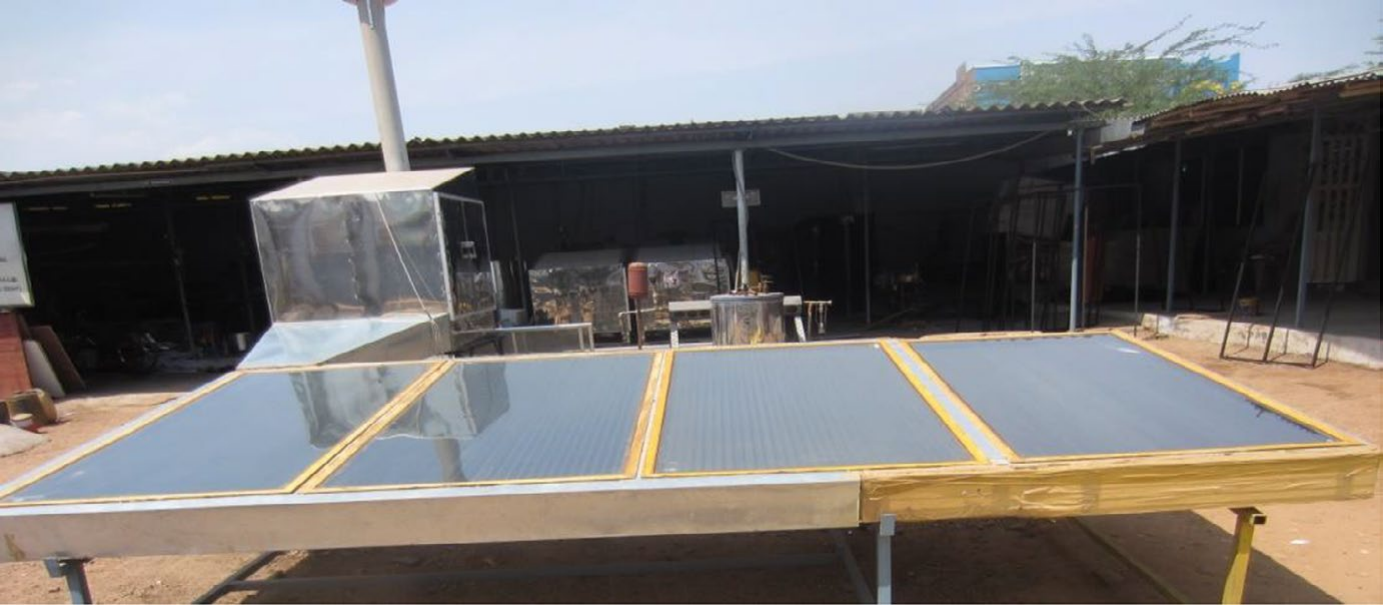
Pictorial representation of the stainless steel solar dryer (SSSD)

The study was carried out in two different locations. The rhizome was subjected to three drying methods, namely tent‐like concrete solar drying (CSD) (Figure 1), which was done at Ghana Atomic Energy Commission, Accra Ghana; stainless steel solar drying (SSSD) (Figure 2) performed at Theni, Tamil Nadu, India; and open‐sun drying (OSD) in India Institute of Food Processing Technology (IIFPT) of Ministry of Food Processing, India (MOFPI), Thanjavur, Tamil Nadu. The CSD sample was couriered to IIFPT laboratories in India and analyzed together with the SSSD and OSD samples.

### 2.3 Research Design

The effects of three treatments on the microbial load of rhizomes were assessed as follows:

- One lot of fresh ginger samples was analyzed for microbial load in its raw state as obtained from source and designated "Fresh unwashed" (funw).
- A second lot of fresh sample was washed in potable water and analyzed for microbial load and designated "Fresh washed" (fwa).
- Some of the washed samples in the second lot (B) was cut into slices and submerged in 10% vinegar for 1 hr and analyzed for microbial load: designated "Fresh in vinegar"(fv).

Part of the samples from treatment C (i.e., vinegar‐treated) was divided into three parts; one lot was open‐sun‐dried (OSD), and the remaining two lots were dried using a tent‐like concrete solar dryer (CSD) located in Ghana and stainless steel box dryer (SSSD) in Theni, India, after which the microbial load was determined.

### 2.4 Washing and drying

The fresh ginger was submerged into fresh water for 2 hr to loosen the soil/debris. It was then thoroughly washed using a soft sponge to clear it of all debris before rinsing again with water. The washed ginger rhizome was manually cut into slices of about 3‐5mm thickness using a kitchen knife. The sliced ginger was soaked in 10% vinegar for one (1) hour and then spread on stainless steel drying trays (1m x1m in length and width). Each tray carried approximately 1 kg of sliced ginger. The preprocessing for the CSD was done in Ghana, as well as the drying, while preprocessing for the SSSD and OSD was done in India using the same protocol for all.

## 3 DRYING METHODS

The samples were dried under two different methods, namely open‐sun drying and solar drying as in 2.3C till the moisture content was about 8.0%–12% dwb. The temperature in the solar dryers ranged between 32 and 42^0^C, 44 and 58^0^C, and 25.5 and 36.6 ^0^C for CSD, SSSD, and OSD, respectively. The samples in the CSD dried in 5 days, while the samples in the SSSD dried in 15 hr, and OSD took 3 days to dry.

### 3.1 Milling of ginger samples

The samples (fresh washed or dried ginger) were pulverized into paste and powder, respectively, using a Philips mill (HR 2113/05).

### 3.2 Quantitative estimation of bacterial and fungal population

The bacterial and fungal population of the fresh and dried ginger samples was determined using the spread plate method described in ISO^4833^:, 2003–2 and ISO^21527^:, 2008–1&2, respectively, with slight modifications. Thirty grams of the ginger sample was transferred into 500‐ml conical flasks containing 270 ml of 0.1% peptone water as diluent. Each flask was shaken at 140 rev min‐^1^ for 20 min on an orbital shaker. Serial dilution up to 1:10^9^ was made, and 0.1 ml aliquot was inoculated in sterile petri dishes containing already‐poured and dried Plate Count Agar (PCA) of about 20 ml for bacteria or Dichloran Rose Bengal Chloramphenicol Agar (DRBC Agar) and Dichloran‐18‐Glycerol for fungi. All the PCA plates were incubated at 35°C for 24 hr, and fungal plates were incubated at 28 ± 2°C for 5–7 days. Plates containing bacterial and fungal colonies were counted, and the population was expressed as CFU/g sample.

### 3.3 Determination of *Salmonella sp*. Contamination

This followed the procedure described in ISO^6579^:, 2002. Twenty‐five grams of ground ginger (fresh or dried) was added to 225 ml of peptone water and shaken for 20 min. The culture was incubated at 37°C ± 1°C for 24 ± 3 hr. An aliquot of 0.1 ml of the culture was taken and added to 10 ml of Rappaport–Vassiliadis Soya Peptone (RSV) Broth. The mixture was incubated at 41.5 ± 1°C for 24 ± 3 hr. This culture was then plated on bismuth sulfite agar at 37°C ± 1°C for 24 ± 3 hr.

Colonies with black center and a lightly transparent zone of reddish color or pink with a darker center were subcultured in Nutrient Broth and incubated at 37°C ± 1°C for 24 ± 3 hr. A little of the incubated culture was then stabbed and streaked on TSI agar and incubated at 37°C ± 1°C for 24 ± 3 hr. A red slant and yellow butt indicated the presence of *Salmonella sp*. Results were expressed as presence or absence of *Salmonella sp*.

### 3.4 Preparation of ginger extracts for vegetative growth analysis

Forty grams of pulverized fresh in vinegar (fv) and dried ginger samples was weighed into a 2‐L conical flask and mixed with 1 L of distilled water until a homogenous mixture resulted. This was allowed to stand for two hours. The aqueous ginger extract was filtered using Whatman's No. 1 filter paper. Thirty milliliters of the aqueous extract was dispensed into 100‐mL Erlenmeyer flasks and sterilized by autoclaving at 1.1kg/ steam pressure at a temperature of 121°C for 15 min. The same was done for 40 g of Potato Dextrose Broth (PDB), which was used as a positive control.

### 3.5 Estimation of vegetative growth of selected fungal isolates

The aqueous ginger extract and PDB (30 ml) in triplicates were inoculated with 1‐cm fungal mycelium block. The Erlenmeyer flasks were incubated for 7 days at 28–30°C. Growth in the liquid medium was assessed by estimating the dry weight of the harvested mycelium. This was done by draining the content of the flasks on a previously oven‐conditioned and weighed Whatman No. 1 filter paper. The filter paper containing the mycelium was dried at 70°C for 5 hr and then reweighed after cooling in a desiccator. The difference in weight was used as the amount of growth.

### 3.6 Identification of fungi

Fungal colonies that developed after the incubation period were counted and identified using cultural and morphological characteristics as outlined by Sampson and Van Reene‐Hoekstra (1988).

### 3.7 Statistical Analysis

One‐way analysis of variance (ANOVA) test at *p* ≤ .05 was used to determine significant differences between treatments for bacterial and fungal load in triplicates using the JMP statistical software (from SAS). The pairwise comparison among means was done using Tukey–Kramer HSD test.

## 4 RESULTS AND DISCUSSION

### 4.1 Initial microbial load of fresh and dried yellow ginger rhizome

For an agricultural product such as a rhizome, the microbial load depends on the sanitary and microbial conditions of the soil it is in contact with. Table 1 shows the data for bacterial and fungal load of the ginger samples before and after treatment. The unwashed samples recorded bacterial load Too Numerous To Count (TNTC) with a population of 2,180 x 10^9^ ± 70.7 x 10^9^ CFU/g, which was significant to all the other treatments as shown in Table 1. There were no significant differences in the microbial load of samples washed in water (fwa) or washed then soaked in 10% vinegar (fv).

**Table 1:** Initial microbial load of fresh and dried ginger using different solar drying methods

Type of sample	Treatment	Type of microorganism (CFU/g) Bacteria Fungi		*Presence of Salmonella sp*
Fresh ginger	Unwashed (funw)	2,180 x 10^9^ ± 70.7 x 10^9 a^	2.50 x 10^4^ ± 2.83 x 10^3 a^	Not detected
	Washed (fwa)	35.0 x 10^9^ ± 7.07 x 10^9 b^	1.20 x 10^4^ ± 2.83 x 10^3 b^	Not detected
	Vinegar (fv)	30.0 x 10^9^ ± 0.00 ^b^	6.00 x 10^3^ ± 1.41 x 10^3 b^	—
Dried ginger	Stainless steel solar‐dried	3.00 x 10^8^ ± 1.41 x 10^8 b^	3.00 x 10^2^ ± 1.41 x 10^2 c^	—
	Concrete solar‐dried	5.1 x 10^7^ ± 1.41 x 10^7 b^	3.00 x 10^3^ ± 1.41 x 10^3 c^	—
	Open‐sun‐dried	1.10 x 10^9^ ± 7.07 x 10^7 b^	5.00 x 10^3^ ± 7.07 x 10^2bc^	—

Note

Values are expressed as mean ± *SEM* of triplicate analysis. Different alphabets as superscript within the same column denote statistical differences (*p* ≤ .05)

not analyzed

The bacterial load of samples showed trends, indicating that washing reduced the microbial load and not the use of 10% vinegar, which could be attributed to loss of soil debris that contained microorganisms. This corroborates reports by Pruthi (1992) that washing prior to processing is desirable to remove field contaminants (dust, soil). There were only marginal changes in the bacterial load when samples were washed and then soaked in 10% vinegar, and this could be attributed to the fact that the pH of 10% vinegar was still a favorable medium for the survival of the bacteria. Similar load of bacteria as high as 9.35 ± 1.76 x 10^9^ was recorded by Mendi et al. (2009) of washed fresh ginger rhizomes.

Exposure to temperature in the solar dryers and open‐sun drying did not significantly reduce the bacterial load of the samples when compared to washing in vinegar. The bacterial population of the solar‐ and sun‐dried samples was in a range of 5.1 × 10^7^–1.10 × 10^9^ CFU/g, which is similar to the findings of Mckee (1995), recording a bacterial population greater than 10^7^ CFU/g of 54 dried spices including ginger, and Mendi et. al. (2009), showing that oven‐dried ginger powder harbored a bacterial population of 7.31 ± 0.03 × 10^6^. This level of contamination is too high to be considered as safe. The OSD samples had bacterial populations almost as high as the "fv" samples due to uncontrolled environment showing the least reduction among the drying methods. This agrees with Eze and Agbo (2011) who showed in their work that the microbial load of solar‐dried ginger was slightly better than that of the open‐sun‐dried.

According to the Codex Alimentarius Commission (1995), spices should not contain S*almonella sp*. *Salmonella* species have been associated with both human and animal illnesses, and it has been one of the most commonly reported causes of human foodborne diseases (Mead et al., 1999). This microorganism was absent from the fresh sample used for the study. The absence of *salmonella sp* from the ginger samples reduces or clears the risk associated with its infection.

The fungal load of the fwa ginger of 1.20 x 10^4^ ± 2.83 x 10^3^ agrees with Li et al. (2017) who showed that fresh fruits, vegetables, and spices such as green pepper, spinach, tomatoes, and cucumber contain fungal population in the range of 2.0 × 10^3^ to 1.0 × 10^4^(3.2 ± 0.9–4.1 ± 0.6 log_10_) CFU/g. There was a significant difference (*p* ≤ .05) between the fungal load of the fresh washed and all the dried samples. The fungal load of the fwa (1.20 × 10^4^ ± 2.83 × 10^3^) samples reduced as compared to the "fv" (6.00 × 10^3^ ± 1.41 × 10^3^), but this was not statistically significant. This could possibly mean that certain associated fungi could still survive the acidity of the 10% vinegar treatment. Unlike the pattern of the bacterial load, the different design of the two solar dryers (Figures 1 and 2) significantly reduced the fungal load as shown in Table 1. On the other hand, the differences in the fungal load of the two solar dryers were not significantly different; open‐sun‐dried samples had higher fungal load than solar‐dried samples as in the bacterial load. Even though the drying time was less in the SSSD, CSD had slightly lower bacterial load than SSSD but higher fungal load.

### 4.2 Fungal species isolated from the fresh and dried ginger rhizomes

Six fungal species belonging to 4 genera *(Aspergillus, Penicillium, Mucor, and Rhizopus)* were isolated from both fresh and dried ginger samples (Table 2). Out of the 6 species, the genus *Aspergillus* was the most predominant. This observation agrees with Geeta and Reddy (1990), Tainter and Grenis (1993, 1994), Rani et.al. (1995), Chourasia (1995), Aziz et. al. (1998), Abdulkadir et. al. (2002), Farid et. al. (2013), Jeswal and Kumar (2015), and Haruna et. al. (2016) who isolated among other genera, *Aspergillus* and *Penicillium* as the most predominant.

**Table 2:** List of fungal species isolated from the fresh and dried ginger rhizomes

Fugal species		Type of treatment				
	Fresh unwashed (funw)	Washed (fw)	Vinegar‐treated (fv)	Stainless steel solar‐dried	Concrete solar‐dried	Open‐sun‐dried
*A. fumigatus*	+	+	+	‐	‐	‐
*flavus*	+	+	+	+	+	+
*niger*	+	‐	‐	‐	‐	‐
*Mucor sp*.	+	‐	‐	‐	‐	‐
*Rhizopus sp*.	+	‐	‐	‐	‐	‐
*Penicillium sp*	+	+	+	+	+	‐

Note

**Key**

(+)—detected ‐—not detected

*A—Aspergillus*

These two genera are of much importance to food safety because they are mycotoxigenic fungi. There are about 400 known mycotoxins of which aflatoxins are the most prevalent and probably most potent. *Aspergillus flavus, A. parasiticus, and A. nomius* produce aflatoxins, which are potential hepatotoxins, mutagenic, and carcinogenic to humans and of possible toxicity to animals as well (Bircan, 2005; Fernandez‐Cruz et. al., 2010; Haruna et. al., 2016; Khazaeli et al., 2017). According to Haruna et. al. (2016) and Elshafie et al. (2002), spice contamination with aflatoxins occurs in the field, during drying and storage, or when it is being passed through other processes. In this study, the isolation of *A. flavus* in all the raw samples as received from the market to the dried samples indicates that the contamination was from the field that persisted through the processing stages. There is the need for farmers to observe good agricultural practices to meet the safe tolerance levels since mycotoxins are naturally occurring and cannot be totally eliminated without damaging the food product (Fung & Clark, 2004). Apart from *P. citrinum* that is known to produce the mycotoxin citrinin, some species of *Penicillium* are also known to produce mycotoxins such as patulin and ochratoxins. In this study, it was observed that the *Penicillium sp*. survived all the treatment except in open‐sun drying (Table 2). This calls for a better pretreatment method if solar dying is the choice of drying.

The fresh unwashed samples recorded more fungal species than all the other treatments as expected of agricultural products that have been contaminated with soil mycoflora. The fresh samples recorded six fungal species (Table 2). The number of species generally reduced as the ginger was washed, treated with vinegar, and dried (Table 2), culminating in a reduction of not less than 50% (Table 3). This shows that washing and vinegar treatment can reduce fungal load and thereby reduce the incidence of foodborne illnesses associated with ginger.

**Table 3:** Percentage occurrence of fungal species isolated from the fresh and dried ginger rhizomes

Fugal species		Type of treatment				
	Fresh unwashed (funw)	Washed (fw)	10% vinegar‐treated (fv)	Stainless steel solar‐dried	Concrete solar‐dried	Open‐sun‐dried
*A. fumigatus*	37.0 ± 3.7	50.0 ± 8.3	33.3 ± 0.0	—	—	—
*A.flavus*	14.8 ± 3.7	8.3 ± 8.3	22.2 ± 0.0	66.7 ± 33.3	66.7 ± 33.3	100 ± 10.0
*niger*	3.7 ± 3.7	+	—	—	—	—
*Mucor sp*.	3.7 ± 0.0	—	—	—	—	—
*Rhizopus sp*.	3.7 ± 0.0	—	—	—	—	—
*Penicillium sp ^b^*	37.0 ± 3.7	41.7 ± 8.3	44.4 ± 22.2	33.3 ± 0.0	37.0 ± 3.7	—

Note

Key

——not detected Values are expressed as mean of three replicates (*n* = 3).

*A—Aspergillus*

The isolation of *A. fumigatus* in the fresh ginger rhizomes calls for concern because the ingestion of this fungus has been associated with cough, respiratory distress, fever, fatigue, interstitial or alveolar infiltrates, and leukocytosis. The solar‐dried samples did not record *A. Fumigatus* and the sun‐dried samples. This study shows the need for further processing of ginger before consumption since all the fungi isolated are of significant health concern. The two mucorales (*Mucor* and *Rhizopus*) found in the samples are known to cause mucormycosis (Rammaert et. al., 2012). These are opportunistic infections in organ transplant and diabetic mellitus patients (Rammaert et. al., 2012). All the fungi isolated were filamentous fungi with serious health implications to the consumer. The percentage occurrence of the fungi as affected by the different processes is shown in Table 3.

### 4.3 Vegetative growth of selected fungi in the ginger extract

**Figure 3:**
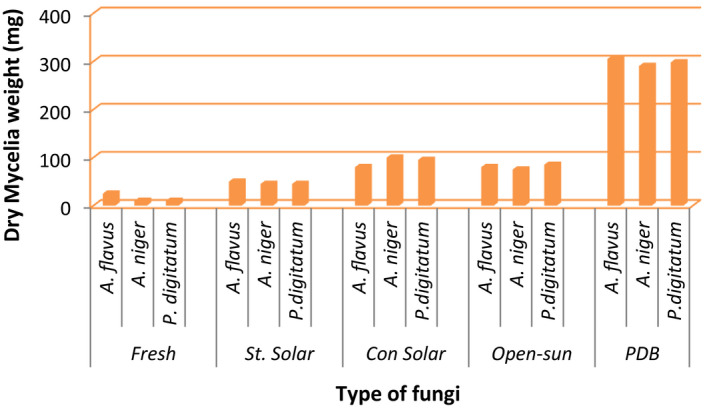
Vegetative growth of selected fungi isolated from the fresh and dried ginger rhizomes as compared to growth in 40g/L of Potato Dextrose Broth (PDB). Fresh—fresh ginger rhizome. St. Solar—stainless steel solar dryer. Con solar—concrete solar dryer. Open‐sun—open‐sun dryer. A—*Aspergillus. P—penicillium*

The ginger extract was used as a growth medium for selected fungi isolated from the fresh and dried ginger samples and compared with growth in PDB. The result as shown in Figure 3 depicts that PDB was ten times a better substrate for fungal growth than the fresh ginger extract and three times better than the dried ginger extract. Thus, the growth of selected fungi was less in the ginger extract as compared to the growth in the PDB.

This could be because the aqueous extract of ginger has antimicrobial properties (Singletary, 2010). The main nutraceutical of functional significance in fresh ginger is gingerol (Cho et. al., 2015; Mohammed et. al., 2019), and it has been shown to have antimicrobial properties. Gingerol is converted to shogaol and zingerone which are less potent in dried ginger and may account for the elevated growth of the fungi in the dried ginger extract as compared to growth in the fresh extract. This suggests that drying decreases the potency of the antimicrobial activity in ginger. All the selected fungi, *A. flavus, A. niger, and P. digitatum,* had higher growth in the dried extract than the fresh samples, suggesting that the antimicrobial activity is higher in the fresh ginger samples than the dried samples. This agrees with reports by Jolad et al. (2004), Balladin et al.(1998), and Zhang et al. (1993, 1994) who showed that the concentrations of gingerols normally found in fresh ginger were reduced but still present on drying. This means that fresh ginger may have more health benefits than the dried ginger, which could be used by the pharmaceutical companies in preparation of antimicrobial drugs.

The study recorded a higher growth of the 3 selected fungi in the extract of the CSD than that of the SSD as shown in Figure 3 with a growth similar to that of OSD, a pattern similar to the fungal growth population (Table 1).

## 5 CONCLUSION

Solar and open‐sun drying of the fresh ginger reduced the bacterial population by twofold counts. That notwithstanding the bacterial population was still significantly high enough to be of food safety concern. The use of 10% vinegar as pretreatment did not show a significant difference (*p* ≤ .05) in bacterial population reduction even though the actual values showed some reduction. The differences in the designs of the two solar dryers did not significantly affect the bacterial load. Conversely, the fungal population showed a significant difference from the samples treated with 10% vinegar to the solar‐dried samples. Stainless steel solar dryer had fewer fungal load than the concrete solar dryer and open‐sun drying. *Salmonella sp*. was not present in the fresh ginger rhizomes. All the six isolated fungal species were filamentous fungi with *Aspergillus* species predominating. This genus is mycotoxin‐producing fungi, which could pose health hazards to consumers. The low mycelia growth of three selected fungal species, namely *A. flavus, A. niger, and P. digitatum,* in the fresh and dried ginger rhizome extract suggests that the ginger rhizome is not a good substrate for fungal growth as compared to growth in Potato Dextrose Broth (PDB). Even though fresh ginger may be contaminated with high microbial population, this study also shows that it may possess some antimicrobial properties against some selected fungi.

## 6 ETHICAL REVIEW

This study does not involve any human or animal testing.

## CONFLICT OF INTEREST

The authors declare that they do not have any conflict of interest.
